# The Oxygen-Generating Calcium Peroxide-Modified Magnetic Nanoparticles Attenuate Hypoxia-Induced Chemoresistance in Triple-Negative Breast Cancer

**DOI:** 10.3390/cancers13040606

**Published:** 2021-02-03

**Authors:** Fong-Yu Cheng, Chia-Hsin Chan, Bour-Jr Wang, Ya-Ling Yeh, Ying-Jan Wang, Hui-Wen Chiu

**Affiliations:** 1 Department of Chemistry, Chinese Culture University, Taipei 11114, Taiwan; zfy3@ulive.pccu.edu.tw; 2 Department of Pharmacological Sciences, Stony Brook University, Stony Brook, NY 11790, USA; chia-hsin.chan@stonybrook.edu; 3 Department of Occupational and Environmental Medicine, National Cheng Kung University Hospital, Tainan 70101, Taiwan; smalladou@mail.cnu.edu.tw; 4 Department of Cosmetic Science and Institute of Cosmetic Science, Chia Nan University of Pharmacy and Science, Tainan 71710, Taiwan; 5 Department of Environmental and Occupational Health, College of Medicine, National Cheng Kung University, Tainan 70101, Taiwan; linn7627@mail.ncku.edu.tw; 6 Department of Medical Research, China Medical University Hospital, China Medical University, Taichung 40402, Taiwan; 7 Graduate Institute of Clinical Medicine, College of Medicine, Taipei Medical University, Taipei 11031, Taiwan; 8 Division of Nephrology, Department of Internal Medicine, Shuang Ho Hospital, Taipei Medical University, New Taipei City 23561, Taiwan; 9 TMU Research Center of Urology and Kidney, Taipei Medical University, Taipei 11031, Taiwan; 10 Department of Medical Research, Shuang Ho Hospital, Taipei Medical University, New Taipei City 23561, Taiwan

**Keywords:** hypoxia, nanocarriers, chemoresistance, triple-negative breast cancer, autophagy

## Abstract

**Simple Summary:**

Tumor hypoxia is known to increase the resistance of cancer cells to chemotherapy. Triple-negative breast cancer (TNBC) is the most aggressive subtype of breast cancer and lack of target. Therefore, chemotherapy is the only approved systemic treatment in TNBC. Here, we synthesized the calcium peroxide-modified magnetic nanoparticles (CaO_2_-MNPs) with the function of oxygen generation to improve and enhance the therapeutic efficiency of doxorubicin treatment in the hypoxia microenvironment of TNBC. CaO_2_-MNPs promoted ubiquitination and protein degradation of hypoxia-inducible factor 1α (HIF-1α). Furthermore, CaO_2_-MNPs inhibited autophagy and induced apoptosis in TNBC cells. CaO_2_-MNPs in combination with doxorubicin showed a stronger tumor-suppressive effect on TNBC compared to the doxorubicin treatment alone in an orthotopic mouse model. Our findings suggest that combined with CaO_2_-MNPs and doxorubicin attenuates HIF-1α expression to improve the efficiency of chemotherapy in TNBC.

**Abstract:**

Cancer response to chemotherapy is regulated not only by intrinsic sensitivity of cancer cells but also by tumor microenvironment. Tumor hypoxia, a condition of low oxygen level in solid tumors, is known to increase the resistance of cancer cells to chemotherapy. Triple-negative breast cancer (TNBC) is the most aggressive subtype of breast cancer. Due to lack of target in TNBC, chemotherapy is the only approved systemic treatment. We evaluated the effect of hypoxia on chemotherapy resistance in TNBC in a series of in vitro and in vivo experiments. Furthermore, we synthesized the calcium peroxide-modified magnetic nanoparticles (CaO_2_-MNPs) with the function of oxygen generation to improve and enhance the therapeutic efficiency of doxorubicin treatment in the hypoxia microenvironment of TNBC. The results of gene set enrichment analysis (GSEA) software showed that the hypoxia and autophagy gene sets are significantly enriched in TNBC patients. We found that the chemical hypoxia stabilized the expression of hypoxia-inducible factor 1α (HIF-1α) protein and increased doxorubicin resistance in TNBC cells. Moreover, hypoxia inhibited the induction of apoptosis and autophagy by doxorubicin. In addition, CaO_2_-MNPs promoted ubiquitination and protein degradation of HIF-1α. Furthermore, CaO_2_-MNPs inhibited autophagy and induced apoptosis in TNBC cells. Our animal studies with an orthotopic mouse model showed that CaO_2_-MNPs in combination with doxorubicin exhibited a stronger tumor-suppressive effect on TNBC, compared to the doxorubicin treatment alone. Our findings suggest that combined with CaO_2_-MNPs and doxorubicin attenuates HIF-1α expression to improve the efficiency of chemotherapy in TNBC.

## 1. Introduction

Triple-negative breast cancer (TNBC) is a subtype of breast cancer defined by the absence of progesterone receptor, estrogen receptor and human epidermal growth factor receptor 2 [1,2]. TNBC accounts for up to 24% of all newly diagnosed breast cancers [3]. Lack of expression of these proteins makes TNBC an orphan disease when considering standard therapeutic regimens for breast cancer [4]. Current treatments of TNBC often includes surgery, radiotherapy and chemotherapy. Due to lack of target in TNBC, chemotherapy is the only approved systemic treatment [1]. However, cancer response to chemotherapy is regulated not only by intrinsic sensitivity of cancer cells but also by tumor microenvironment. Previous studies have demonstrated that tumor hypoxia, a condition of low oxygen level in solid tumors, increases the resistance of cancer cells to chemotherapy and radiotherapy [5,6]. Hypoxia affected breast cancer cell growth dynamics, angiogenesis, migration, endoplasmic reticulum (ER) stress and aggressive features [7,8]. About 50–60% of advanced solid tumors, including breast cancer, comprise hypoxic region which is often related with poor survival [9,10,11]. Hypoxia-inducible factor 1α (HIF-1α) is an essential regulator of molecular response to hypoxia. HIF-1α is degraded in normoxic conditions. Furthermore, hypoxia increased HIF-1α expression, which in turn changes many gene products that affect the regulation of metabolism, angiogenesis, cell cycle and apoptotic process [12]. Accumulating evidences have shown the involvement of hypoxia in the induction of chemo- and radio-resistance [11,13,14]. In addition, many reports have demonstrated that hypoxia increased chemoresistance in cancer cells through the activation of the HIF-1α pathway [15,16]. These findings reveal that HIF-1α plays a critical role in regulating tumor chemosensitivity. Recent evidence shows that parkin binds to HIF-1α and causes HIF-1α degradation by ubiquitination, which suppresses metastasis of breast cancer cells [17]. The HIF-1α protein degradation in hypoxic conditions is ubiquitination-dependent [18].

Autophagy is a crucial homeostatic process in the human body that is responsible for the elimination of damaged proteins, lipids and organelles [19]. Autophagy is also a significant regulatory pathway during cancer cells against hypoxic stress. Therefore, autophagy is known to increase cancer cells survival and therapy resistance [20]. Cicchini et al. indicated that *BECN1*, which is an autophagy regulator, inhibited mammary tumorigenesis through WNT1 activation [21]. Previous studies have demonstrated that autophagy constrains sensitivity to cytotoxic therapy. Radiotherapy or chemotherapy induced autophagy is accompanied by therapy resistance in breast cancer cells [22]. Another recent study illustrated that hypoxia triggers cytoprotective autophagy and consequently attenuates sensitivity of TNBC cells to taxol treatment [23], suggesting that increasing oxygen tension may be an approach to increase chemotherapy sensitivity.

Nanotechnology has recently gained increased attention for cancer prevention, detection and treatment. Nanoparticles (NPs) have used to drug delivery, allowing for therapeutic drugs to selectively targeting tumor tissue, while reducing toxicity in normal cells [24]. Furthermore, nanocarriers are ideal platform for cancer therapies because they possess the properties of solubility enhancement effects, high drug loading capacity, controlled release and site-specific delivery mechanism [25]. Previous studies have reported using oxygen carrying microbubbles to reduce the tumor hypoxia and enhance sonodynamic therapeutic effect [26,27]. Li et al. showed that co-delivery of erlotinib and oxygen by liposomal complexes reverses hypoxia-induced drug resistance in lung cancer. Moreover, nanotherapeutics containing oxygen adjusts hypoxic microenvironment in tumor to improve therapeutic outcomes [28]. This study used calcium peroxide-modified magnetic nanoparticles (CaO_2_-MNPs) as an oxygen generation substrate because previous reports had indicated CaO_2_ has suitable oxygen release rate and good biocompatibility for bioapplications [29,30,31]. The study showed that oxygen is released from the CaO_2_-MNPs after mixture of CaO_2_-MNPs and water. In the present study, we examine the effect of doxorubicin which is a chemotherapeutic drug in hypoxic conditions in TNBC cells. Furthermore, we evaluate the anti-cancer effects of doxorubicin combined with CaO_2_-MNPs in TNBC cells in vitro and in an orthotopic mouse model. We present data showing that CaO_2_-MNPs treatment in TNBC reduces hypoxic stress in tumor evident by decreased HIF-1α expression and increased doxorubicin-sensitivity in vivo.

## 2. Results

### 2.1. Hypoxia Increases the Resistance of Cancer Cells to Chemotherapy in TNBC

**Figure 1:**
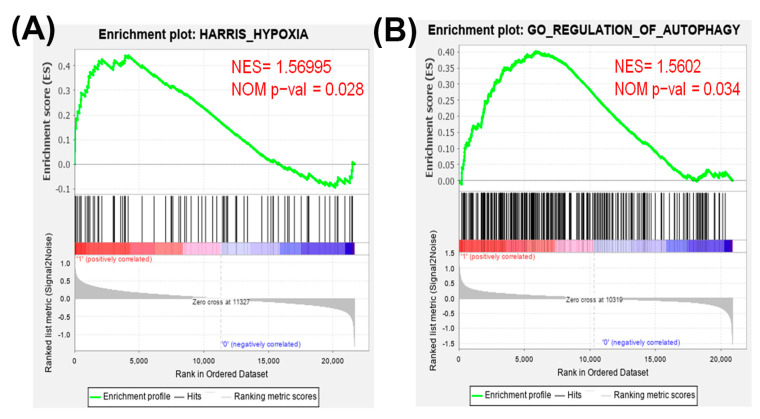
Gene set enrichment analysis (GSEA) of hypoxia and autophagy gene expressions in primary tumors and normal tissues derived from patients with triple-negative breast cancer (TNBC). All of the expressed genes were uploaded into GSEA for enrichment analysis. The Harris hypoxia (**A**) and Gene Ontology (GO) regulation of autophagy (**B**) gene sets database were used as the gene set collection for analysis. NES, normalized enrichment score; NOM p-val, nominal *p* value.

**Figure 2:**
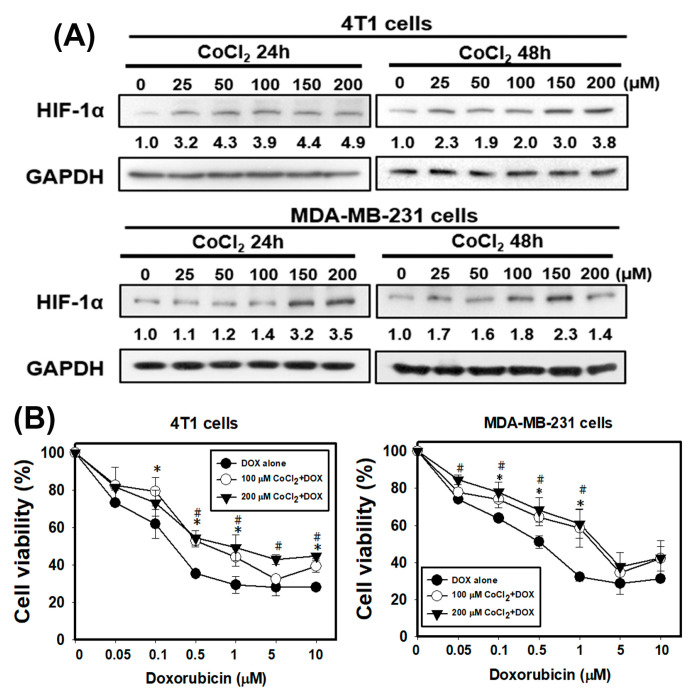
The chemical hypoxia (CoCl_2_) stabilizes hypoxia-inducible factor 1α (HIF-1α) protein and causes doxorubicin (DOX) resistance in TNBC cells. (**A**) the protein level of HIF-1α in 4T1 and MDA-MB-231 cells treated with CoCl_2_ for 24 and 48 h. (**B**) effect of DOX on cell viability of 4T1 and MDA-MB-231 cells in the absence or presence of CoCl_2_. Cells were pretreated with CoCl_2_ for 48 h and then treated with DOX for 24 h. * *p* < 0.05 DOX alone compared with 100 μM CoCl_2_ + DOX. # *p* < 0.05 DOX alone compared with 200 μM CoCl_2_ + DOX.

Firstly, the expression values of all of the genes in 17 normal tissues and primary tumors derived from 33 patients with TNBC were uploaded into gene set enrichment analysis (GSEA) software to analyze the enriched functions within the hypoxia gene set database (Figure 1A). The results identified that the hypoxia gene set was found to be significantly enriched in TNBC patients. Therefore, hypoxia plays an important role in TNBC. Next, we determine whether hypoxic condition increases the doxorubicin-resistance in TNBC cells. Cobalt chloride (CoCl_2_) is a mimetic agent used in vitro to cause cellular hypoxia. CoCl_2_ stabilizes HIF-1α by suppressing prolyl hydroxylase enzymes [32]. We analyzed the expression of HIF-1α protein to confirm that the CoCl_2_ can effectively cause HIF-1α protein stabilization in TNBC cells. As shown in Figure 2A, the treatment with CoCl_2_ increased the expression levels of HIF-1α protein in a concentration-dependent manner in human breast cancer cells MDA-MB-231 and murine breast cancer cells 4T1. We next tested the impact of hypoxia on chemosensitivity. Pre-treatment with CoCl_2_ increased doxorubicin-resistance of MDA-MB-231 and 4T1 cells in a concentration-dependent manner of doxorubicin (Figure 2B). These results found that hypoxia can cause resistance of TNBC cells to doxorubicin.

### 2.2. Hypoxia Inhibits the Induction of Apoptosis and Increases Autophagy by Doxorubicin in TNBC

**Figure 3:**
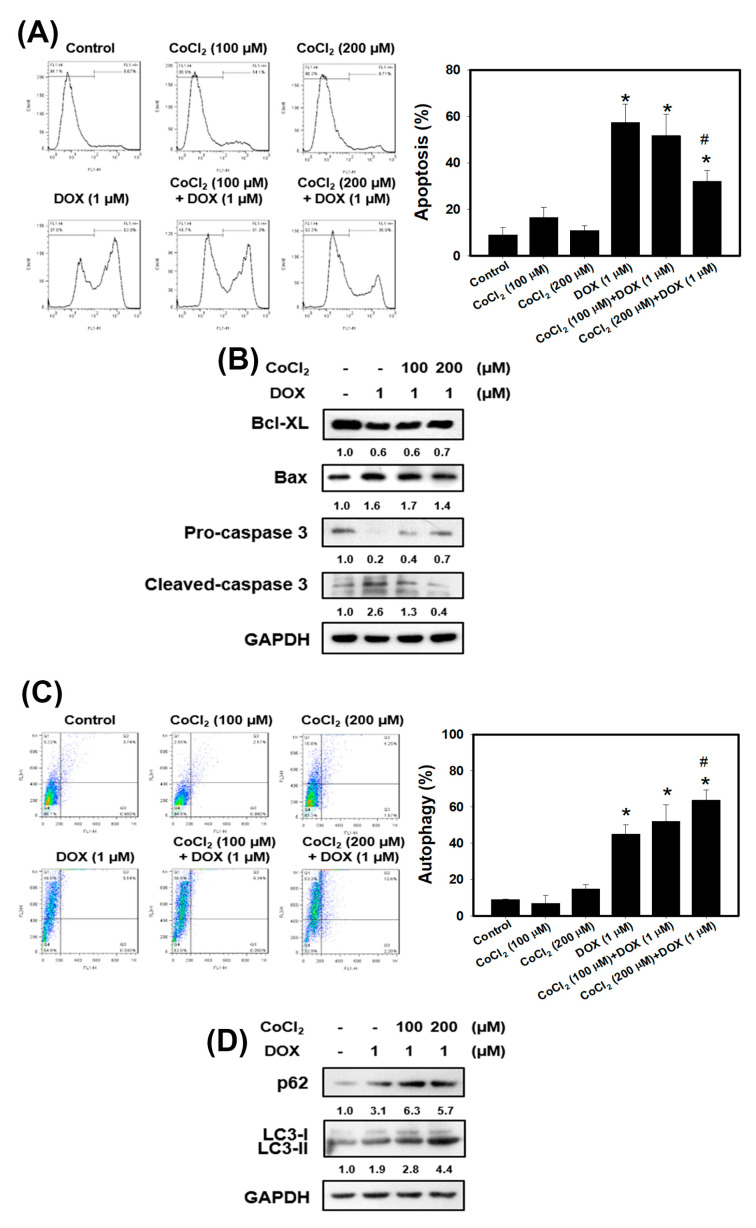
Detection of apoptosis and autophagy in 4T1 cells that received DOX and/or CoCl_2_ treatments. (**A**) apoptosis was analyzed by flow cytometry with an Annexin V apoptosis detection kit. 4T1 cells were pretreated with CoCl_2_ for 24 h and then treated with DOX for 24 h. * *p* < 0.05 compared with control. # *p* < 0.05 DOX alone compared with 200 μM CoCl_2_ + DOX. (**B**) effects of apoptosis-related protein expression in 4T1 cells treated with DOX and CoCl_2_ alone or in combination. Cells were pretreated with CoCl_2_ for 24 h and then treated with DOX (1 μM) for 24 h. (**C**) measurement of acidic vesicular organelles (AVOs) in acridine orange (AO)-stained cells using flow cytometry. 4T1 cells were pretreated with CoCl_2_ for 24 h and then treated with DOX for 24 h. * *p* < 0.05 compared with control. # *p* < 0.05 DOX alone compared with 200 μM CoCl_2_ + DOX. (**D**) effects of autophagy-related protein expression in 4T1 cells treated with DOX and CoCl_2_ alone or in combination. Cells were pretreated with CoCl_2_ for 24 h and then treated with DOX (1 μM) for 24 h.

Previous research has shown that hypoxia promoted radioresistance through suppressing radiation-induced apoptosis in cervical cancer cells [13]. In order to investigate whether hypoxia has a protective effect against apoptosis induced by doxorubicin in TNBC cells, 4T1 cells were treated in the presence or absence of doxorubicin under normoxia or hypoxia. Apoptosis in 4T1 cells was examined using flow cytometry with Annexin V (Figure 3A). The quantitative results indicated that doxorubicin induced approximately 60% of apoptosis. However, 200 μM CoCl_2_ protected 4T1 cells from apoptotic cell death induced by doxorubicin. Next, we detected the apoptosis-related protein expression using Western blot analysis. The expression level of the anti-apoptotic protein (Bcl-XL) was inhibited, and pro-apoptotic protein (Bax) and cleaved-caspase 3 were increased with doxorubicin treatment (Figure 3B). CoCl_2_ enhanced the doxorubicin-inhibited Bcl-XL and suppressed the doxorubicin-induced Bax and cleaved-caspase 3. All these data demonstrate that hypoxia can protect TNBC cells against the well-known apoptosis inducer, doxorubicin. 

The gene expression in normal tissues and primary tumors derived from patients with TNBC were uploaded into GSEA software to evaluate the autophagy gene set database (Figure 1B). The data showed that the autophagy gene set was significantly enriched in TNBC patients. Previously the authors reported that doxorubicin triggered autophagy in breast cancer cells [33]. Moreover, autophagy increases cancer cells survival and therapy resistance [20]. To investigate whether CoCl_2_ can affect autophagy, we used the acridine orange (AO) stain which detected acidic vesicular organelles (AVOs) of autophagy feature [34]. We quantified AVOs in AO-stained cells by flow cytometry (Figure 3C). 4T1 cells treated with doxorubicin showed significantly increased AO-positive cells. Furthermore, cells pre-treated with CoCl_2_ induced higher percentage of autophagic cells than that of the doxorubicin alone, accompanied with upregulation of the autophagy-related proteins p62 and LC3-II (Figure 3C,D).

### 2.3. Chemical and Physical Characteristics of CaO_2_-MNPs

**Figure 4:**
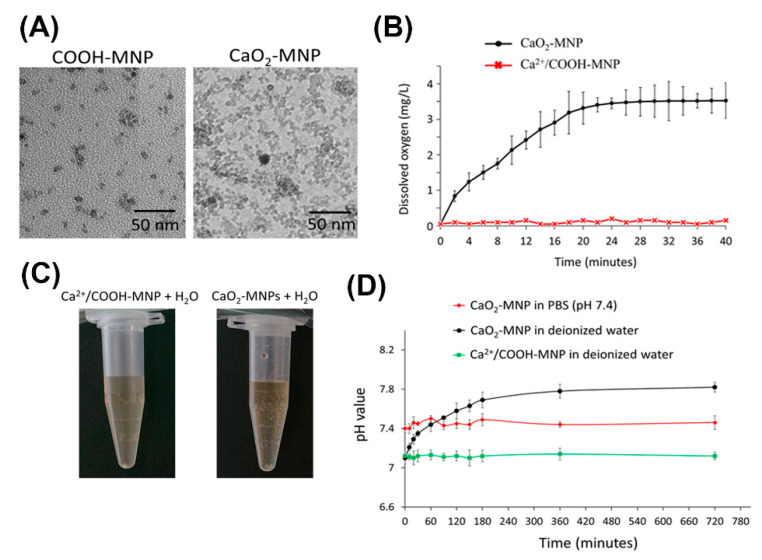
Chemical and physical characteristics of calcium peroxide-modified magnetic nanoparticles (CaO_2_-MNPs). (**A**) TEM images of COOH-modified MNPs (COOH-MNPs) and CaO_2_-MNPs. The used solvents for preparing TEM samples of COOH-MNPs and CaO_2_-MNPs were water and polyethylene glycol (M.W. 200) (PEG200), respectively. (**B**) dissolved oxygen kinetics after the addition of Ca^2+^/COOH-MNPs or CaO_2_-MNPs (0.1 mL; Fe concentration: 0.9 mg/mL) into 20 mL of water at room temperature. (**C**) optical photographs of Ca^2+^/COOH-MNPs and CaO_2_-MNPs incubated with water. (**D**) the pH value profiles after addition of Ca^2+^/COOH-MNPs and CaO_2_-MNPs (0.1 mL; Fe concentration: 0.9 mg/mL) in 20 mL of PBS buffer (pH 7.4) or water at room temperature.

The CaO_2_-MNPs were successfully prepared from amine group-modified MNPs (NH_2_-MNPs) via two states of COOH-modified MNPs (COOH-MNPs) and Ca^2+^/COOH-MNPs. TEM images of COOH-MNPs and CaO_2_-MNPs are shown in Figure 4A and their real diameters of COOH-MNPs and CaO_2_-MNPs are calculated about 7.1 nm and 7.3 nm. The hydrodynamic diameters of COOH-MNPs and CaO_2_-MNPs are 32.5 nm and 45.1 nm measured by dynamic light scattering (DLS) instrument (Figure S1A). The surface charges of COOH-MNPs and CaO_2_-MNPs are −30.6 mV and −9.3 mV (Figure S1B). Dissolved oxygen (DO) release profile of CaO_2_-MNPs in the close system was monitored over time (Figure 4B). The elevating DO levels of CaO_2_-MNPs was near to 3.5 mg/L, whereas the Ca^2+^/COOH-MNPs (precursor of CaO_2_-MNPs) had no oxygen production in water. Optical photographs showed that bubbles (oxygen) were produced after CaO_2_-MNPs mixed with water for 10 min (Figure 4C), but no bubbles could be observed in Ca^2+^/COOH-MNPs mixed with water at the same time interval. Furthermore, the pH value varieties of CaO_2_-MNPs in PBS buffer (pH 7.4) and deionized water were measured by pH meter (Figure 4D). The slightly increased and no changed pH values were separately observed when CaO_2_-MNPs incubated with deionized water and PBS buffer. No changes of pH values were also found when Ca^2+^/COOH-MNPs mixed with water.

### 2.4. CaO_2_-MNPs Promote Ubiquitination and Protein Degradation of HIF-1α, and Increases TNBC Sensitivity to Doxorubicin in Hypoxic Conditions

**Figure 5:**
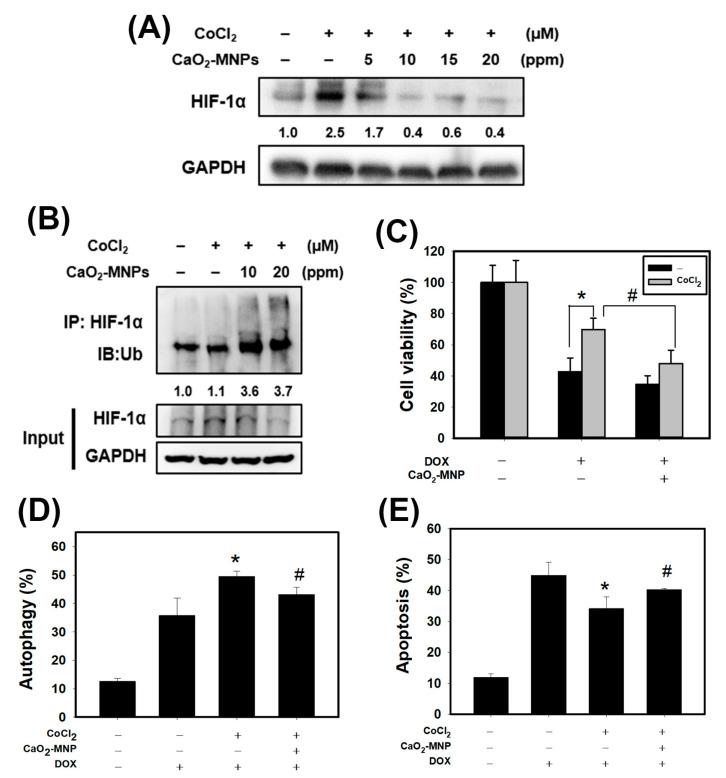
CaO_2_-modified MNPs cause protein degradation and ubiquitination of hypoxia-inducible factor 1α (HIF-1α), and reverts the effects of DOX on apoptosis and autophagy in 4T1 cells. (**A**) Western blot analysis for HIF-1α protein expression. Cells were incubated with CoCl_2_ (100 μM) for 24 h, treated with CaO_2_-MNPs for 24 h, then harvested for protein extraction and then subjected to western blot analysis. (**B**) immunoprecipitation (IP) assay for HIF-1α ubiquitination. 4T1 cells incubated with CoCl_2_ (100 μM) for 24 h and treated with CaO_2_-MNPs for 24 h. The total protein lysates harvested from 4T1 cells were immunoprecipitated with anti-HIF-1α antibody. The complexes and the input were examined using western blot analysis with anti-ubiquitin antibody. Effect of CaO_2_-MNPs on cell viability (**C**), autophagy (**D**) and apoptosis (**E**) of 4T1 cells. Cells were pretreated with CoCl_2_ (100 μM) for 8 h, treated with CaO_2_-MNPs (20 ppm) for 16 h, then incubated with DOX (1 μM) for 24 h. * *p* < 0.05 DOX alone compared with DOX + CoCl_2_. # *p* < 0.05 DOX + CoCl_2_ compared with DOX + CoCl_2_ + CaO_2_-MNPs.

We found that CaO_2_-modified MNPs can inhibit the CoCl_2_-stabilized HIF-1α in a concentration-dependent manner in 4T1 cells (Figure 5A). It is well known that rapid degradation of specific proteins by ubiquitin-proteasome system (UPS) is a component of many cellular mechanisms [35]. We speculated that CaO_2_-MNPs cause HIF-1α degradation through ubiquitination. To study this possibility, we investigated the HIF-1α ubiquitination by immunoprecipitation (IP). We observed that CaO_2_-MNPs induced HIF-1α ubiquitination (Figure 5B). Therefore, CaO_2_-MNPs-triggered the HIF-1α degradation may be regulated by UPS. Next, we found that pre-treatment with CoCl2 markedly increased doxorubicin-resistance in 4T1 cells (Figure 5C). However, CaO_2_-MNPs inhibited the CoCl_2_-induced doxorubicin-resistance. We further investigated the effect of autophagy and apoptosis by CaO_2_-MNPs. CoCl_2_ increased the doxorubicin-induced autophagy while CaO_2_-MNPs significantly suppressed that effect (Figure 5D). Additionally, CaO_2_-MNPs reverted doxorubicin-induced apoptosis under CoCl_2_ treatment (Figure 5E).

### 2.5. The Anti-Cancer Effects of Doxorubicin in Combination with CaO2-MNPs in an Orthotopic Mouse Model

**Figure 6:**
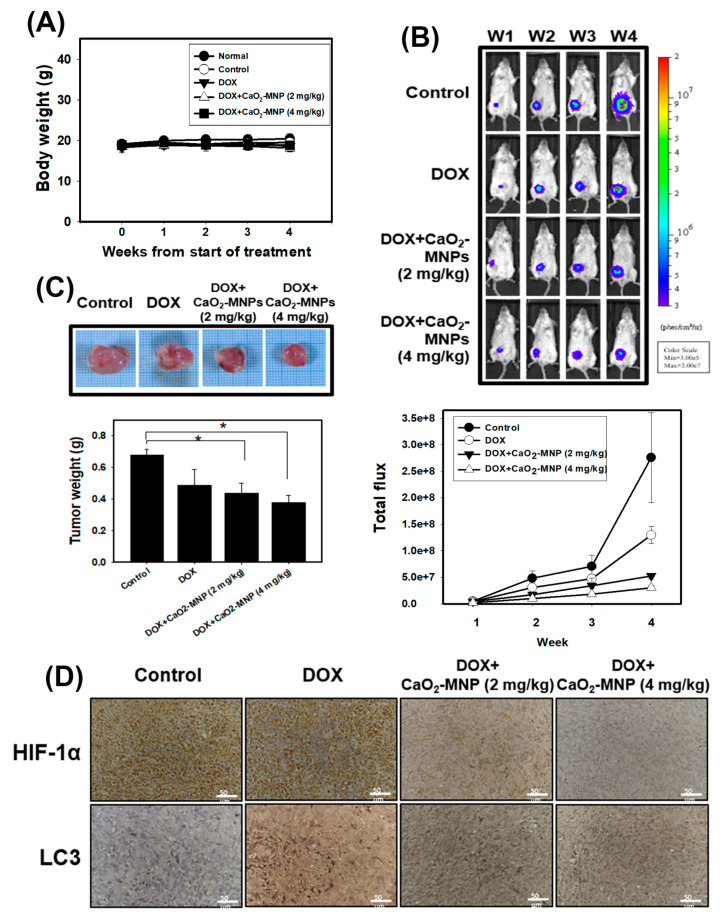
The anti-cancer effects of DOX in combination with CaO_2_-MNPs in an orthotopic mouse model. The female Balb/c mice were injected with 5 × 10^4^ 4T1 cells orthotopically into the 4th mammary fat pad. (**A**) the body weight was measured weekly in the different groups. (**B**) the bioluminescence imaging of tumors was used in vivo imaging system (IVIS) 200 system with Living Image Software. (**C**) the tumor weight was analyzed after sacrificing. * *p* < 0.05 control compared with DOX + CaO_2_-MNPs. (**D**) specific immunostaining was conducted on 4T1 tumor sections showing the expression of HIF-1α and LC3 in response to various treatments in vivo. Scale bars = 50 µm.

4T1 cells were stably transfected with luciferase (4T1-Luc) and 4T1-Luc cells were used in the orthotopic breast cancer of mouse model. 4T1-Luc cells were inoculated into the mammary fat pads of Balb/c mice. Mice were observed for 4 weeks following treatment with doxorubicin (DOX) and CaO_2_-MNPs. The body weights of mice were determined and none of the treatment produced significant losses of body weight or any apparent abnormalities (Figure 6A). Furthermore, tumor growth was monitored weekly with an in vivo imaging system (IVIS) system and quantified via bioluminescence imaging (Figure 6B). The results showed that tumor volume had a gradual increase in the control group. We found while DOX alone decreased primary growth of TNBC, CaO_2_-MNPs further enhanced TNBC response to DOX in vivo (Figure 6B). We also observed a significant reduction in tumor weight in mice received the combination treatment in comparison to those treated with DOX alone (Figure 6C). In addition, we performed histological staining of tumor sections with antibodies against HIF-1α and LC3, the readouts of hypoxia and autophagy, respectively (Figure 6D). HIF-1α expression in tumor tissue in the combination groups was lower than that in the control and DOX groups. Moreover, DOX in combination with CaO_2_-MNP further decreased LC3 expression compared to the DOX treatment alone. These results indicated that the combination of DOX and CaO_2_-MNP inhibited autophagy and hypoxia in vivo and thus suppressed tumor growth in an orthotopic TNBC model.

## 3. Discussion

Accumulating evidence has reported that many advanced solid tumors may display anoxic and/or hypoxic tissue areas that are heterogeneously distributed within the tumor mass. The oxygenation level in several cancers including breast cancer is found to be lower than that in the respective normal tissues [11]. In the present study, the results from GSEA uncovered that the hypoxia gene set was significantly enriched in TNBC tissues (Figure 1A). When a tumor is growing, some regions of tumors undergo a decrease in O_2_, growth factors and glucose due to the poor vascularization of these regions [36,37]. Hypoxia increases HIF-1α expression and the mechanisms by HIF-1α target genes led to poorer patient survival through decreasing apoptosis, enhancing cancer cell survival and induction of angiogenesis [11]. Previous research has shown that the Bcl-2 family anti-apoptotic member Mcl-1 was overexpressed by hypoxia in human polymorphonuclear leukocytes [38]. Hypoxia can also protect cells against apoptotic cell death induced by anti-cancer drugs (taxol and fluorouracil) in pheochromocytoma cells [39]. In this study, we showed the evidence that chemical hypoxia (CoCl_2_) stabilized HIF-1α protein (Figure 2A) and reduced cell death induced by doxorubicin in 4T1 cells (Figure 2B). Furthermore, hypoxia inhibited doxorubicin-induced apoptosis and the expression of apoptosis-associated proteins (Figure 3).

CaO_2_-MNPs were successfully prepared and mainly confirmed by increased hydrodynamic diameters (Figure S1A) and obvious changes of surface charges (Figure 1B). The obvious change in surface charges from −30.6 mV (from COOH-MNPs) to −9.3 mV due to Ca^2+^ adsorbed the COO¯ groups on the particle surface to form Ca^2+^/COOH-MNPs (Figure S1B). The increased hydrodynamic diameter from 32.5 nm to 45.1 nm also confirmed the successful synthesis of CaO_2_-MNPs from COOH-MNPs. TEM images showed CaO_2_-MNPs were slightly aggregated (Figure 4A). Actually, CaO_2_-MNPs showed the well-dispersion and no aggregation in water because of its hydrodynamic diameter was 45.1 nm (Figure S1B). To evaluate the oxygen release function of CaO_2_-MNPs, CaO_2_-MNPs and Ca^2+^/COOH-MNPs were mixed with deoxygenated water at room temperature. The obvious increased DO values were observed in CaO_2_-MNPs and the elevating DO levels was 3.5 mg/L (Figure 4B). These results proved that oxygen was certainly generated from CaO_2_-MNPs. Notably, the bubbles were only found when CaO_2_-MNPs incubated with water for 10 min (Figure 4C). No bubbles could not be observed when Ca^2+^/COOH-MNPs incubated with water, even the incubation time prolonged to 2 h. To further evaluate the safety of CaO_2_-MNPs for further cellular and animal experiments, the pH values were monitored after CaO_2_-MNPs incubated with PBS buffer (pH 7.4) and deionized water (Figure 4D). Because the side products after CaO_2_-MNPs mixed with water were Ca(OH)_2_ and water. Ca(OH)_2_ could produce OH¯ to increase pH value in water. As seen as Figure 4D, the pH values slightly increased from 7.0 to 7.8 when CaO_2_-MNPs mixed with deionized water for about 5 h, but the pH values kept about 7.4 when CaO_2_-MNPs mixed with PBS buffer (pH 7.4). This means CaO_2_-MNPs will not affect or change the pH value in physiological system after they are injected into body. Notably, when Ca^2+^/COOH-MNPs incubated with deionized water, the pH values also had no changes. These results confirmed that CaO_2_-MNPs could generate oxygen again.

Ample evidence has indicated that the poor vascular blood and flow oxygen availability in hypoxic tumor tissues caused insufficient drug delivery [40]. Currently, nanocarriers has great potential to specifically enhance drug accumulation in hypoxic tumor cells due to enhanced permeability and retention (EPR) effect [41]. Previously the authors reported that nanoparticles co-loading oxygen and erlotinib reversed hypoxia-induced drug resistance in lung cancer. This drug and oxygen co-delivery strategy downregulated the expression of the protein p-EGFR, EGFR and HIF-1α, markedly suppressed cell proliferation and triggered apoptosis [28]. Another study concluded that the oxygen-delivery microbubbles decreased the hypoxia of pancreatic cancer cells and ameliorated sonodynamic therapeutic effect [26]. In the current study, we used CaO_2_-MNPs to release oxygen and found that CaO_2_-MNPs can degrade the CoCl_2_-stabilized HIF-1α protein in TNBC cells (Figure 5A). Moreover, CaO_2_-MNPs decreased the HIF-1α expression in tumor tissues of TNBC (Figure 6D). Oxygen by CaO_2_-MNPs can induce HIF-1α ubiquitination and cause HIF-1α degradation (Figure 5B). However, more precise molecular mechanism of HIF-1α ubiquitination by oxygen should be investigated in the future. In addition, CaO_2_-MNPs inhibited the CoCl_2_-induced doxorubicin-resistance (Figure 5C) but promoted the doxorubicin-induced apoptosis under CoCl_2_ treatment (Figure 5E). Moreover, an orthotopic TNBC mouse model showed a significantly decreased tumor weight in mice with the combination treatment (CaO_2_-MNPs + DOX) compared to those treated with DOX alone (Figure 6C). Therefore, the combination of DOX and CaO_2_-MNPs decreases HIF-1α protein expression in vivo and, thus, attenuates hypoxia responses to inhibit tumor growth in TNBC.

Our results found that the autophagy gene set was remarkably enriched in TNBC patients (Figure 1B). In our in vitro study, chemical hypoxia (CoCl_2_) itself did not trigger autophagy. However, upon DOX treatment in hypoxic condition TNBC cells did appear to activate autophagy. Furthermore, cells pre-treated with CoCl_2_ induced higher percentage of autophagic cells than that with DOX treatment alone; moreover, this phenotype was, accompanied with upregulation of the autophagy-related proteins p62 and LC3-II (Figure 3C,D). The in vitro and in vivo findings collectively evidenced that combining DOX with CaO_2_-MNP significantly suppressed autophagic effect compared to the DOX treatment alone (Figure 5D and Figure 6D). These observations are consistent with previous reports. In a study conducted in the human TNBC cell line MDA-MB-231, taxol treatment under hypoxic condition activated cytoprotective autophagy through inhibition of the mTOR pathway but hypoxia itself did not induce autophagy. The resistance against taxol-induced cell death under hypoxia can be explained by autophagic process [23]. Wu et al. indicated that autophagy inhibition effectively increased cisplatin-induced apoptosis, suggesting the involvement of autophagy in cisplatin resistance under hypoxia in lung cancer cells [42]. Many studies concluded that autophagy reduces the sensitivity to cancer therapy [20,43]. One key mechanism underlying radiotherapy and chemotherapy-induced resistance is the AMPK pathway, which both activate ULK1 and inhibit mTOR signaling to trigger autophagy [44]. AMPK–ULK1 pathway were found to have a cytoprotective mechanism against chemotherapy in pancreatic cancer cells [45]. Accumulating evidence indicates that many types of cancer cells can activate autophagy to confer resistance to different chemotherapies.

The limitation of the study is the process of oxygen generation of CaO_2_-MNPs. Because the mechanism of oxygen release is due to CaO_2_ reacts with water. It means that CaO_2_-MNPs can immediately generate oxygen when environment has water. In this study, this limitation was very small because CaO_2_-MNPs were injected into mice by intratumorally injection. In future studies, CaO_2_-MNPs will be designed to inject into mice by tail vein injection. Before CaO_2_-MNPs accumulate in tumor region, the oxygen release of CaO_2_-MNPs is continuous. So, the real oxygen amount in tumor region will be less than theoretical value and hard to be estimated. Although this problem can be overcome by increase dosage of CaO_2_-MNPs, the safety drug dosage needs to be concerned if drug-loaded CaO_2_-MNPs are used. The excess drugs may cause side effects. The other limitation of the study is moisture content in tumor region. Unfortunately, the moisture contents in tumor microenvironment are different and hard to estimated. Using the same dosage of CaO_2_-MNPs may generate different amount of oxygen, even the tumor sizes are the same. Therefore, the therapeutic efficiency may have a little difference in experimental results with the same conditions. Importantly, the difference did not affect the proof-of concept that the oxygen release of CaO_2_-MNPs could certainly improve oxygen level in tumor region and then reduce the drug resistance of cancer cells to get more therapeutic efficiency.

## 4. Materials and Methods

### 4.1. Preparation of COOH-Modified MNPs (COOH-MNPs)

To synthesize COOH-MNPs, the NH_2_-MNPs [46] were used as a starting material. First, 1 mL of succinic acid (45 mM), 45 μmol of 1-ethyl-3-(3-dimethylaminopropyl)carbodiimide (EDC) and 9 mL of NH_2_-MNPs (Fe concentration: 2 mg/mL) were mixed for 10 min. Following, the mixture solution stirred for 2 h. Acetone was added the solution to produce precipitates and then precipitates were collected by centrifuged at 6000 rpm for 15 min. The supernatant was discarded, and then deionized water was added to disperse the precipitates (COOH-MNPs) for further use.

### 4.2. Preparation of CaO_2_-Modified MNPs (CaO_2_-MNPs)

The preparation of CaO_2_ referred as described by Khodaveisi et al. [47] with slight modification. First, 1 mL of CaCl_2_ aqueous solution (0.01 M) was added into the aqueous solution of COOH-MNPs and then using a permanent magnet to isolate the precipitates (Ca^2+^/COOH-MNPs). Following, polyethylene glycol (M.W. 200) (PEG200) was added to dissolve the precipitates and the mixtures were sonicated until the precipitates fully dispersed in PEG200. Finally, 2 mL of 30% (*w*/*w*) H_2_O_2_ was slowly added into PEG solution. The mixtures were gently stirred at room temperature for 0.5 h and then centrifuged at 6000 rpm to remove PEG solution. Acetone was used to wash the precipitates twice and then PEG200 was added to redisperse the precipitate (CaO_2_-MNPs) for further experiments. The Fe ion concentration for the CaO_2_-MNPs was determined by an atomic absorption (AA) analysis.

### 4.3. Characterization of CaO_2_-MNPs

The preparation of CaO_2_ referred as described by Khodaveisi et al. [47] with slight modification. First, 1 mL of CaCl_2_ aqueous solution (0.01 M) was added into the aqueous solution of COOH-MNPs and then using a permanent magnet to isolate the precipitates (Ca^2+^/COOH-MNPs). Following, PEG200 was added to dissolve the precipitates and the mixtures were sonicated until the precipitates fully dispersed in PEG200. Finally, 2 mL of 30% (*w*/*w*) H_2_O_2_ was slowly added into PEG solution. The mixtures were gently stirred at room temperature for 0.5 h and then centrifuged at 6000 rpm to remove PEG solution. Acetone was used to wash the precipitates twice and then PEG200 was added to redisperse the precipitate (CaO_2_-MNPs) for further experiments. The Fe ion concentration for the CaO_2_-MNPs was determined by an AA analysis.

### 4.4. Dissolved Oxygen (DO) Kinetics of CaO_2_-MNPs

A measurement method was built by covering a beaker with a form cap. Two holes were cut into the foam cap in order to insert a DO meter and a glass pipette for nitrogen purging. Before the start of each experiments, 20 mL of deionized water was added to the beaker at room temperature, a DO meter was inserted into and purged with nitrogen to remove DO. Then, 1 mL of materials (CaO_2_-MNPs and Ca^2+^/COOH-MNPs) at 0.9 mg/mL (Fe concentration) was injected into the closed system and then the DO values were monitored by a DO meter.

### 4.5. Measuring pH Values in Aqueous Solution of CaO_2_-MNPs

First, 20 mL of deionized water or PBS buffer (pH 7.4) was added to the beaker at room temperature and then a pH meter was inserted into this beaker. Following, 1 mL of CaO_2_-MNPs (or Ca^2+^/COOH-MNPs) at 0.9 mg/mL (Fe concentration) was injected into beaker and then the pH values were monitored by a pH meter.

### 4.6. Gene Set Enrichment Analysis (GSEA)

Microarray data were obtained from the Gene Expression Omnibus (GEO) GSE61725 data set. The gene lists of Harris or GO gene signature were obtained from the Molecular Signatures Database (MSigDB) [48].

### 4.7. Cell Culture

The human breast cancer cell line MDA-MB-231 (ATCC HTB-26) and murine breast cancer cell line 4T1 (ATCC CRL-2539) were purchased from the American Type Culture Collection (ATCC) (Manassas, VA, USA). The luciferase-expressing 4T1-Luc was acquired from Professor Yi-Ching Wang (Department of Pharmacology, National Cheng Kung University, Tainan, Taiwan). The cells were incubated in Dulbecco’s modified essential medium (DMEM) (Gibco BRL, Grand Island, NY, USA) with 100 U/mL of penicillin, 100 μg/mL of streptomycin (Gibco BRL) and 10% fetal bovine serum (Caisson Labs, Logan, UT, USA). The cells were cultured at 37 °C in a humidified atmosphere containing 5% CO_2_. 

### 4.8. Cell Viability

Cells were collected and resuspended in phosphate-buffered saline. Each cell suspension and an equal amount of trypan blue solution was mixed. Then, the mixture was added on a hemocytometer. Alive cells were shown as no trypan blue-stained cells.

### 4.9. Determination of Apoptosis

An Annexin V apoptosis detection kit (Calbiochem, San Diego, CA, USA) was used to assess the phosphatidyl serine translocated to the cell surface. Cells were harvested and were added to Annexin V-FITC. Then, the labeled cells were quantified by flow cytometry (BD Biosciences, San Jose, CA, USA).

### 4.10. Determination of Autophagy

Cell staining with acridine orange (Sigma-Aldrich, St. Louis, MO, USA) was analyzed for detection of acidic vesicular organelles, which is a characteristic of autophagy. Cells were collected and stained with 1 μg/mL AO for 20 min. Then, cells were quantified using flow cytometry (BD Biosciences).

### 4.11. Western Blot Analysis and Immunoprecipitation (IP)

Cells were lysed in lysis buffer at 4 °C for 1 h. Proteins, which were isolated from the cells were loaded and separated by SDS-PAGE. The gels were transferred to polyvinylidene fluoride membranes and the membranes were then blocked with skim milk. The membranes were incubated with primary antibody at 4 °C overnight. An anti-HIF-1α antibody was obtained from Novus Biologicals (Littleton, CO, USA); anti-ubiquitin antibody was obtained from BioLegend Inc. (San Diego, CA, USA); anti-GAPDH, anti-LC3, anti-Bax and anti-Bcl-XL antibodies were obtained from Cell Signaling Technology (Ipswich, MA, USA); active + pro caspase-3 antibody was obtained from ABclonal Inc. (Boston, MA, USA); and an anti-p62/SQSTM1 antibody was obtained from MBL (Nagoya, Japan). After washing three times, the membranes were incubated with horseradish peroxidase (HRP)-conjugated secondary antibody for 1 h. Finally, the signals of membranes were examined by immobilon Western Chemiluminescent HRP Substrate (Merck Millipore, Darmstadt, Germany) and exposed to X-ray film. 

For IP analysis, whole-cell protein lysates were added with anti-HIF-1α antibody overnight at 4 °C and then were added with protein G plus/protein agarose (Merck Millipore, Darmstadt, Germany) for 1 h. After washing, the beads were resuspended and boiled for 10 min at 95 °C. The supernatant was used to western blot analysis as described above. Raw data of Western blot is shown in Figure S2.

### 4.12. In Vivo Orthotopic Breast Cancer Model

Six-week-old female Balb/c mice were obtained from the National Laboratory Animal Center (Taiwan) and housed with 60% ± 5% relative humidity at 23 ± 2 °C in the Laboratory Animal Center, College of Medicine, National Cheng Kung University. The animal experiment was approved by the Institutional Animal Care and Use Committee of National Cheng Kung University (IACUC Approval Number: 107247). 4T1-Luc cells (5 × 10^4^ cells) were injected into the 4th of mammary fat pads in mice for one week. Then, the mice were randomized into four groups (*n* = 5 for each group): (1) the control, which was intraperitoneally (i.p.) injected with DMSO, (2) the DOX group: Which was i.p. injected with 3 mg/kg doxorubicin once per week for three weeks, (3) the DOX+ 2 mg/kg CaO_2_-MNPs group, which was i.p. injected with 3 mg/kg doxorubicin once per week and intratumorally injected 2 mg/kg CaO_2_-MNPs two times per week for three weeks and (4) the DOX+ 4 mg/kg CaO_2_-MNPs group, which was i.p. injected with 3 mg/kg doxorubicin once per week and intratumorally injected 4 mg/kg CaO_2_-MNPs two times per week for three weeks. Mouse body weight was examined once per week. Before imaging, mice were injected with VivoGlo Luciferin (150 mg/kg) (Promega, Madison, WI, USA) and anesthetized with isoflurane. The bioluminescence signal of tumors was used IVIS 200 system with Living Image Software (Xenogen, Alameda, CA, USA). Mice were sacrificed via CO_2_ and tumor tissues were collected and soaked by formalin.

### 4.13. Immunohistochemical (IHC) Staining Analysis

After soaking by formalin and embedding paraffin, tumor sections were deparaffinized in xylene and rehydrated by graded concentrations of ethanol in water. Then, microwave treatment in sodium citrate retrieved antigens. The tumor sections were immersed in 3% H_2_O_2_/methanol for 10 min to inhibit the activity of endogenous peroxidase. After washing, the slides were added with anti-HIF-1α (Novus Biologicals, Littleton, CO, USA) and anti-LC3 (MBL, Nagoya, Japan) antibodies and used a Starr Trek Universal HRP detection kit (Biocare Medical, Concord, CA, USA) to detect the antibodies. Finally, the slides were stained with hematoxylin and coverslipped.

### 4.14. Statistical Analysis

All data are shown as the mean ± standard deviation (SD) of the results from three independent experiments. The statistics of results was used with one-way analysis of variance followed by post hoc Dunnett’s test or two-sample *t*-test. *p* values < 0.05 (*p* < 0.05) in all analyses were considered statistical significance. 

## 5. Conclusions

**Figure 7:**
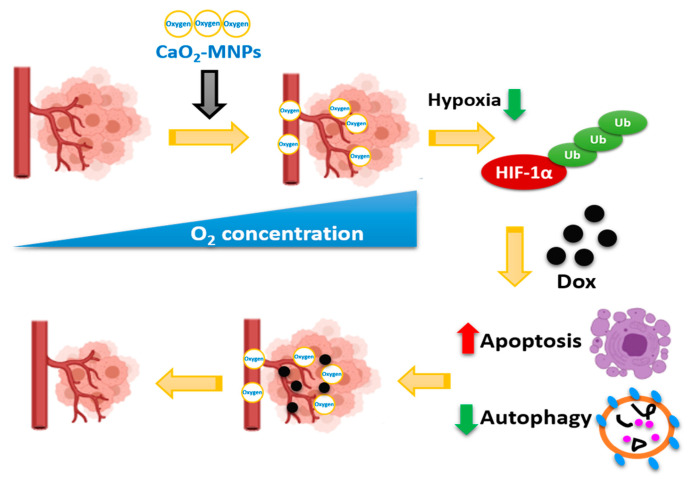
CaO_2_-MNPs attenuate hypoxia-induced chemoresistance in TNBC. CaO_2_-MNPs release oxygen and degrade HIF-1α protein by ubiquitination. Hypoxia inhibit the DOX-induced apoptosis and DOX-increased autophagy in TNBC cells. CaO_2_-MNPs promote cell death against DOX-induced resistance under hypoxia through the inhibition of autophagy and induction of apoptosis. Therefore, co-treatment with CaO_2_-MNPs and DOX could be an effective approach to attenuate HIF-1α-mediated hypoxic responses to modulate TNBC. The figure was created with BioRender.com.

The hypoxia and autophagy gene sets were significantly enriched in TNBC patients, suggesting hypoxia and autophagy play a critical role in TNBC. Furthermore, we showed that DOX induced apoptosis and autophagy activation in TNBC cells. CoCl_2_ increased the resistance of TNBC cells to DOX. Additionally, hypoxia inhibited the induced apoptosis and increased autophagy by DOX. CaO_2_-MNPs can release oxygen and degrade HIF-1α protein by ubiquitination. Moreover, CaO_2_-MNPs promoted cell death against DOX-induced resistance under hypoxia likely through the inhibition of autophagy and induction of apoptosis (Figure 7). In vivo, we found CaO_2_-MNPs further decrease tumor growth and tumor weight under DOX treatment. The in vitro and in vivo findings indicated that the co-treatment of CaO_2_-MNPs and DOX could be an effective approach to diminish HIF1α-mediated hypoxic response to modulate tumor growth of TNBC.

## Data Availability

The data presented in this study are available in article or supplementary material.

## Supplementary Materials

The following are available online at https://www.mdpi.com/2072-6694/13/4/606/s1, Figure S1: Physical characteristics of COOH-MNPs and CaO_2_-MNPs, Figure S2: Raw data of Western blot.
