# Supplementary material for: The Oxygen-Generating Calcium Peroxide-Modified Magnetic Nanoparticles Attenuate Hypoxia-Induced Chemoresistance in Triple-Negative Breast Cancer
Source: Cancers (Basel). 2021 Feb 3;13(4):606. doi: 10.3390/cancers13040606 (PMC7913619; doi:10.3390/cancers13040606)
Supplement: Supplementary file 1 [file cancers-13-00606-s001.pdf]

cancers

MDPI

Supplementary Materials: The Oxygen-Generating Calcium Peroxide-Modified Magnetic Nanoparticles Attenuate Hypoxia-Induced Chemoresistance in Triple-Negative Breast Cancer

Fong-Yu Cheng, Chia-Hsin Chan, Bour-Jr Wang, Ya-Ling Yeh, Ying-Jan Wang and Hui-Wen Chiu

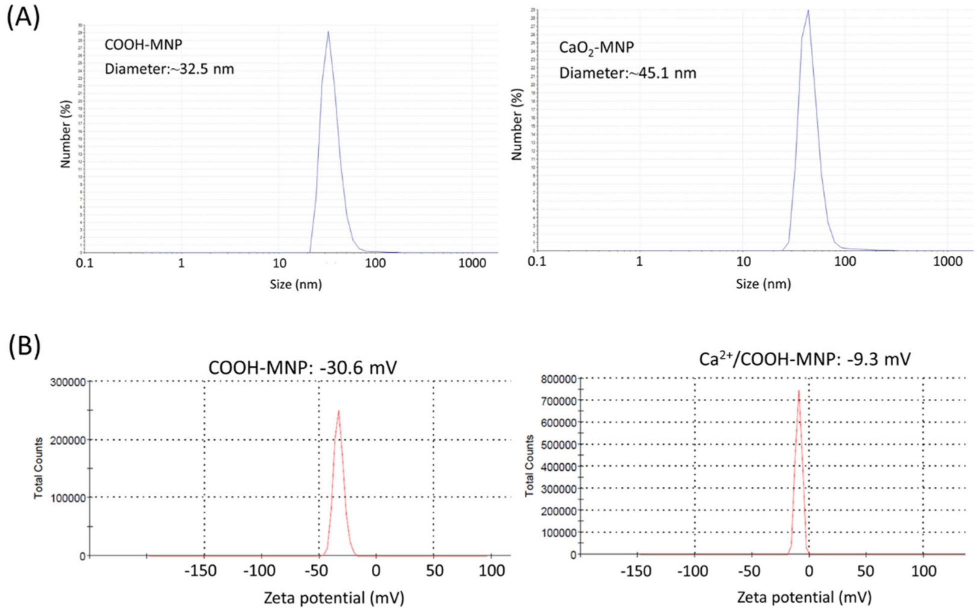

Figure S1. Physical characteristics of COOH-MNPs and CaO_2-MNPs. (**A**) The hydrodynamic diameters of COOH-MNPs and CaO_2-MNPs. (**B**) Zeta potential of Ca^{2+}/COOH-MNPs and CaO_2-MNPs.

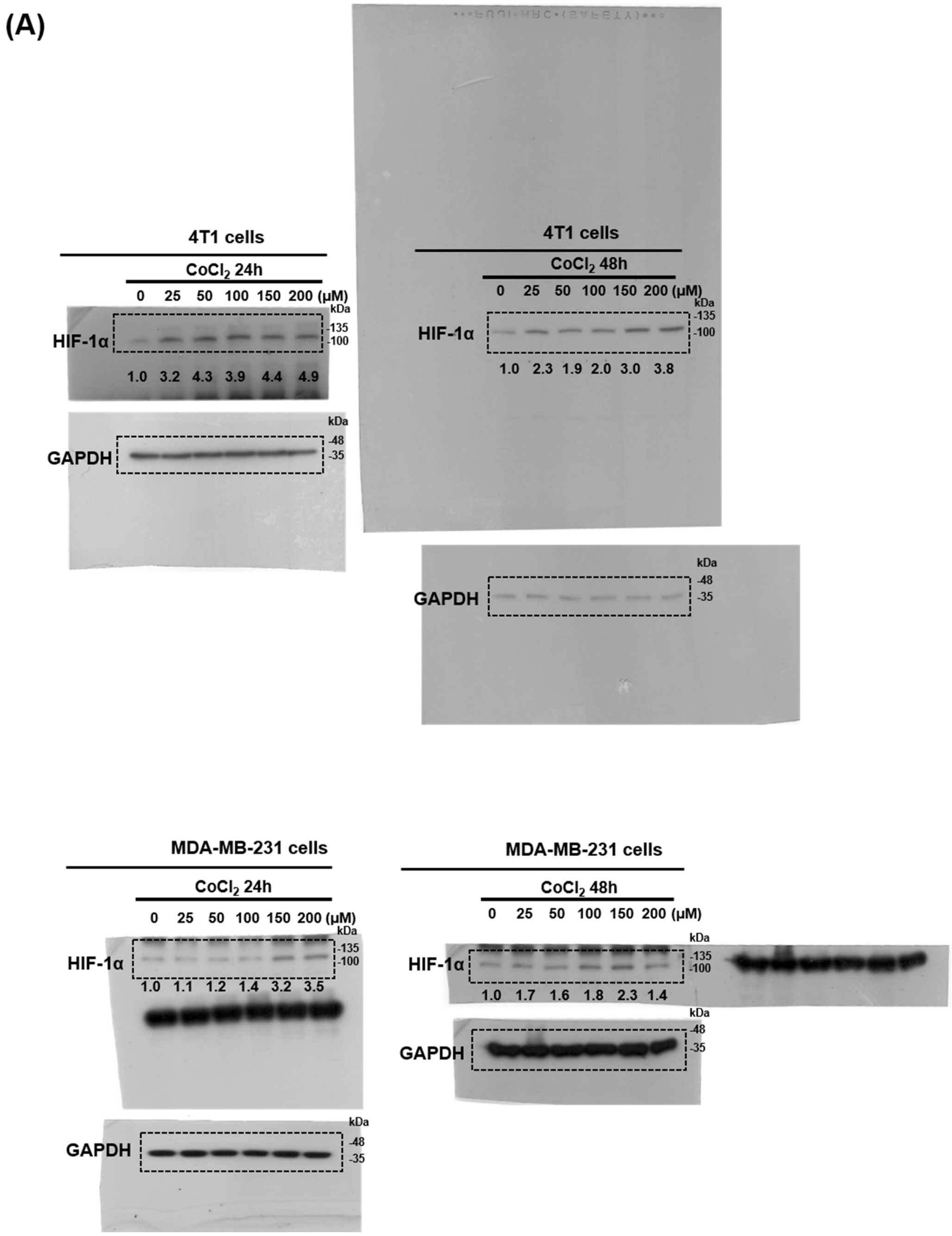
(A)
4T1 cells
CoCl2 24h
0 25 50 100 150 200 (μM)
kDa
HIF-1α
-135
-100
1.0 3.2 4.3 3.9 4.4 4.9
kDa
GAPDH
-48
-35
4T1 cells
CoCl2 48h
0 25 50 100 150 200 (μM)
kDa
HIF-1α
-135
-100
1.0 2.3 1.9 2.0 3.0 3.8
kDa
GAPDH
-48
-35
MDA-MB-231 cells
CoCl2 24h
0 25 50 100 150 200 (μM)
kDa
HIF-1α
-135
-100
1.0 1.1 1.2 1.4 3.2 3.5
kDa
GAPDH
-48
-35
MDA-MB-231 cells
CoCl2 48h
0 25 50 100 150 200 (μM)
kDa
HIF-1α
-135
-100
1.0 1.7 1.6 1.8 2.3 1.4
kDa
GAPDH
-48
-35

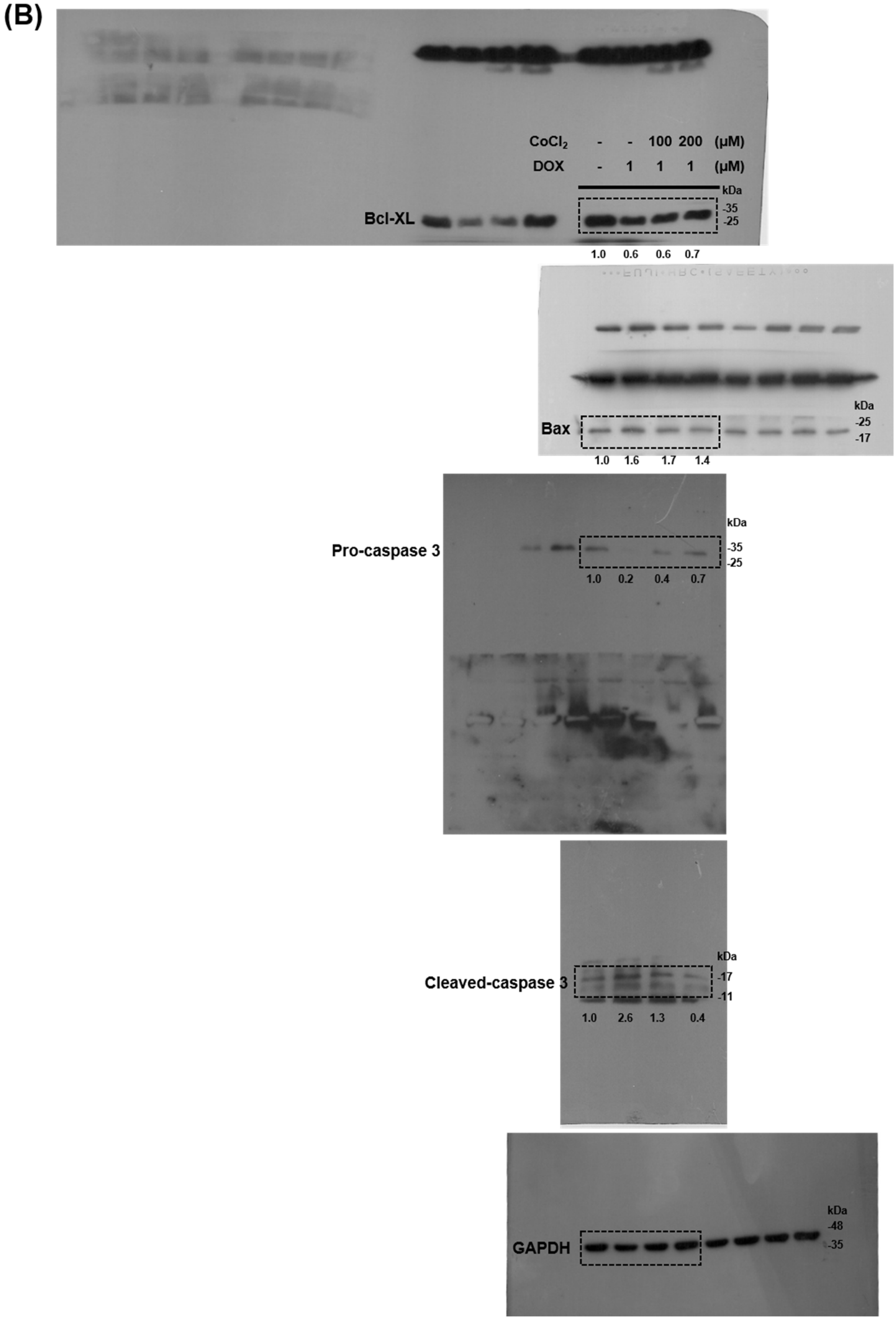
(B)
CoCl2 - - 100 200 (μM)
DOX - 1 1 1 (μM)
kDa
Bcl-XL
-35
-25
1.0 0.6 0.6 0.7
Bax
kDa
-25
-17
1.0 1.6 1.7 1.4
Pro-caspase 3
kDa
-35
-25
1.0 0.2 0.4 0.7
Cleaved-caspase 3
kDa
-17
-11
1.0 2.6 1.3 0.4
GAPDH
kDa
-48
-35

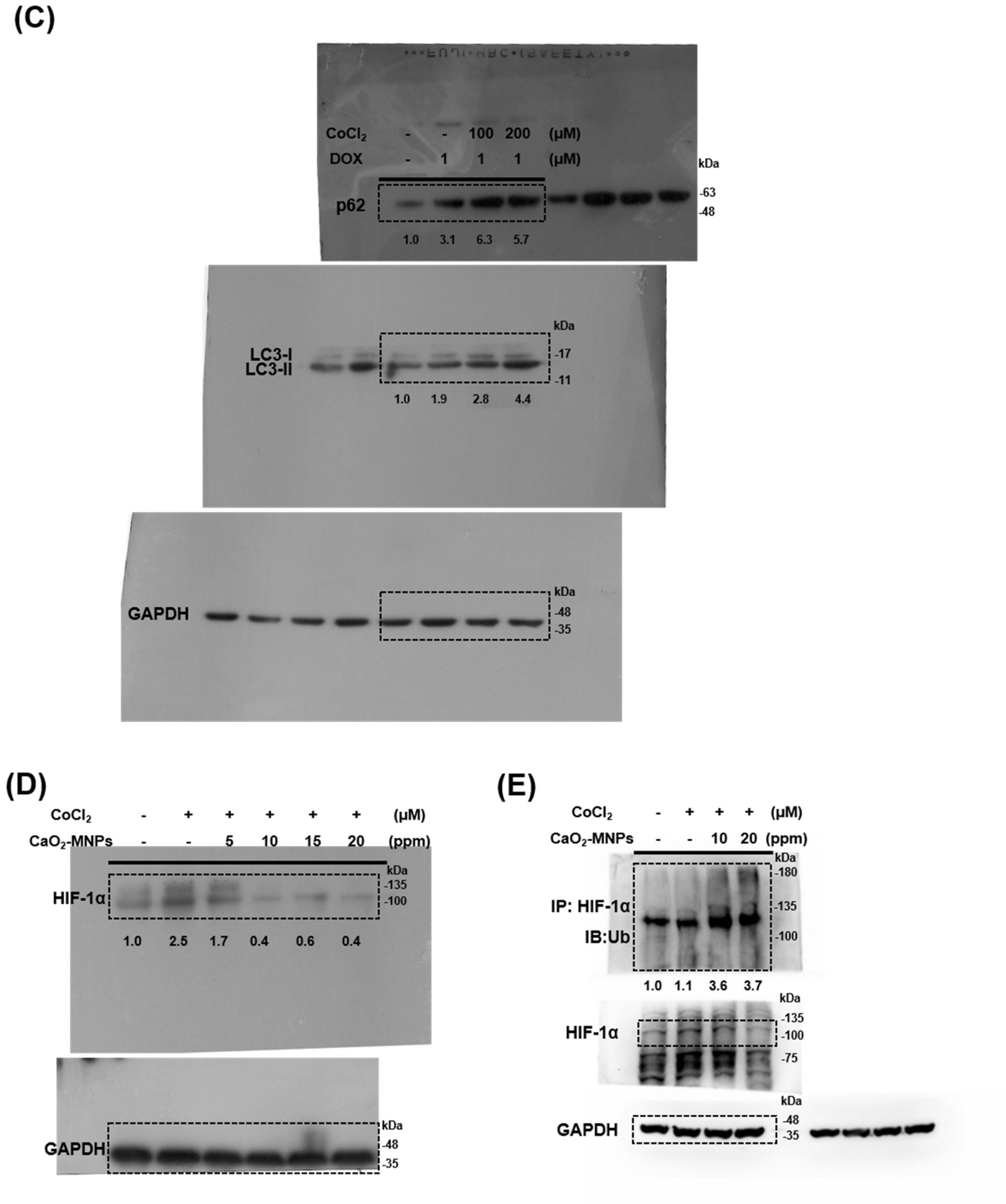

Figure S2. Raw data of Western blot. (**A**) Raw data for figure 2A. (**B**) Raw data for figure 3B. (**C**) Raw data for figure 3D. (**D**) Raw data for figure 5A. (**E**) Raw data for figure 5B.
